# The effect of time-of-day and chest physiotherapy on multiple breath washout measures in children with clinically stable cystic fibrosis

**DOI:** 10.1371/journal.pone.0190894

**Published:** 2018-01-10

**Authors:** Christian Voldby, Kent Green, Susanne Rosthøj, Thomas Kongstad, Lue Philipsen, Frederik Buchvald, Marianne Skov, Tania Pressler, Per Gustafsson, Kim G. Nielsen

**Affiliations:** 1 CF-Centre Copenhagen, Paediatric Pulmonary Service, University of Copenhagen, Copenhagen, Denmark; 2 Research Unit, Women’s and Children’s Health, University of Copenhagen, Copenhagen, Denmark; 3 Department of Biostatistics, Faculty of Health Sciences, Institute of Public Health, University of Copenhagen, Copenhagen, Denmark; 4 Department of Paediatrics, Central Hospital, Skövde, Sweden; Telethon Institute for Child Health Research, AUSTRALIA

## Abstract

**Background:**

In this pilot study we investigated daytime variation of multiple breath nitrogen washout (N_2_MBW) measures in children with clinically stable cystic fibrosis. To our knowledge the effect of time-of-day on multiple breath washout measures in patients with cystic fibrosis has not previously been reported. Furthermore, we assessed the influence of chest physiotherapy on N_2_MBW measures.

**Methods:**

Ten school children with cystic fibrosis performed N_2_MBW followed by spirometry and plethysmography in the morning and afternoon at three visits that were one month apart. Chest physiotherapy was performed immediately before the afternoon measurements at visit 2 and immediately before morning and afternoon measurements at visit 3. The influence of time-of-day and chest physiotherapy on the measures was evaluated using linear mixed models.

**Results:**

There were adequate quality data from 8 children with median age (range) 9.6 (6.0; 15.1) years. Baseline lung clearance index (LCI) (range) was 9.0 (7.1; 13.0) and baseline FEV_1_% predicted was 97.5 (78.5; 117.9). No N_2_MBW measures were significantly influenced by time-of-day or chest physiotherapy. LCI (95% confidence interval) decreased non-significantly 0.05 (-0.32; 0.22) during the day and increased non-significantly 0.08 (-0.26; 0.42) after chest physiotherapy. All spirometric measures were unaffected by time-of-day and chest physiotherapy. For plethysmographic measures FRC_pleth_ decreased significantly (p<0.01) 110 mL during the day, whereas a borderline significant (p = 0.046) decrease in ΔFRC_pleth-MBW_ during the day and a borderline significant (p = 0.03) increase in TLC after CPT were observed.

**Conclusion:**

This study demonstrated that the time-of-day as well as chest physiotherapy performed immediately prior to N_2_MBW had no consistent or significant influence on N_2_MBW measures. However, we emphasize that further studies of the effect of both daytime variation and the effect of chest physiotherapy on multiple breath washout measures are warranted.

## Introduction

The lung clearance index (LCI) as measured by multiple breath washout (MBW) reflects ventilation distribution inhomogeneity (VI) and peripheral airway disease. Due to a high sensitivity to early lung disease in patients with cystic fibrosis (CF) compared to conventional lung function parameters such as FEV_1_[1–3], MBW is increasingly used in research studies and in the clinical management of CF worldwide.

Valid interpretation of comparative and repeated MBW measurements is important and requires knowledge of test variability and factors that influence MBW measures. There are no previous reports of daytime variation of MBW measures in patients with CF. Additionally, the effect of chest physiotherapy (CPT) on these measures has been inconsistent and unpredictable in both children and adults with CF in a limited number of studies[4–6]. Previous studies have shown that mucociliary clearance is low during sleep in healthy subjects[7] and in stable asthmatic patients[8]. Moreover, CPT is part of daily CF treatment and is known to increase expectorated secretions[9–11]. Hence, daytime variation in mucus clearance in CF patients and CPT performed daily could affect MBW measures. Knowledge about daytime variation and the effect of CPT on MBW is thus relevant for designing future studies, for clinical application, and for interpreting MBW results. Moreover, such data are important when evaluating the potential role of MBW in the surveillance of progression in CF lung disease and for assessing treatment effects.

In this pilot study we investigated whether the time-of-day and CPT influenced multiple breath nitrogen washout (N_2_MBW) measures in school children with CF using standard spirometric and plethysmographic parameters as comparators. We hypothesized that daytime clearance of mucus that had built up at night and performing CPT immediately prior to N_2_MBW, resulting in the mobilization of sputum, would improve N_2_MBW measures.

## Materials and methods

### Study population

**Fig 1:**
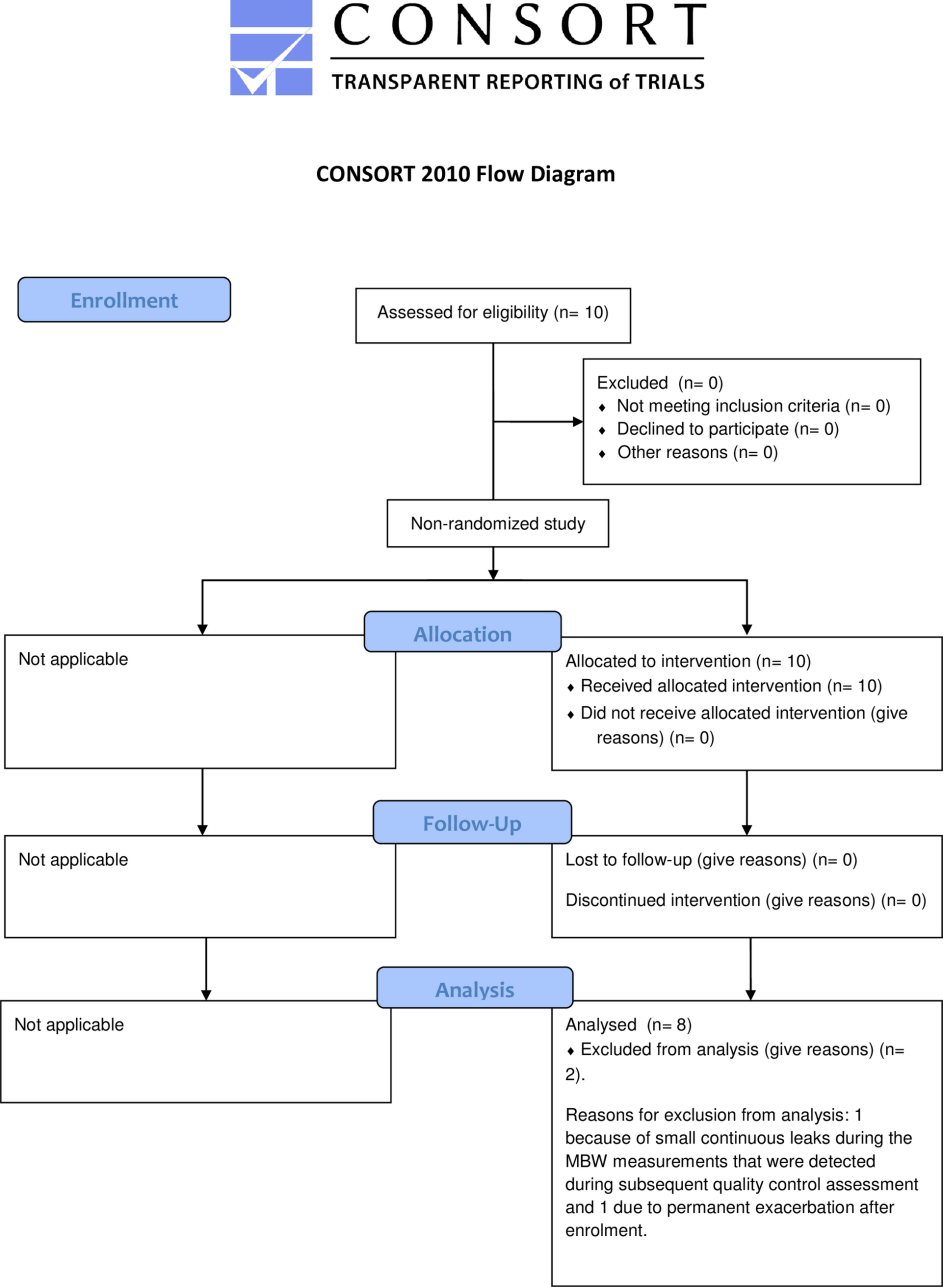
Study flow diagram using the CONSORT 2010 standard.

A total of 10 school children with CF who were between 6–18 years old were recruited from the Paediatric Pulmonary Service, Copenhagen University Hospital, Denmark (Fig 1). Patients were eligible for the study if they were able to perform N_2_MBW and spirometry. Patients had to be clinically stable at all visits according to a modified version of the original Fuchs criteria[12]. Thus, visits were postponed for 1 month if the patient showed signs of an acute or ongoing airway infection with more than 2 of the following 6 symptoms: increased coughing; increased mucus or change in mucus color; malaise, fatigue or lethargy; increased dyspnea; loss of appetite or weight loss; decrease in predicted FEV_1_ more than 10 percentage points from the average of the previous year.

### Study design

**Fig 2:**
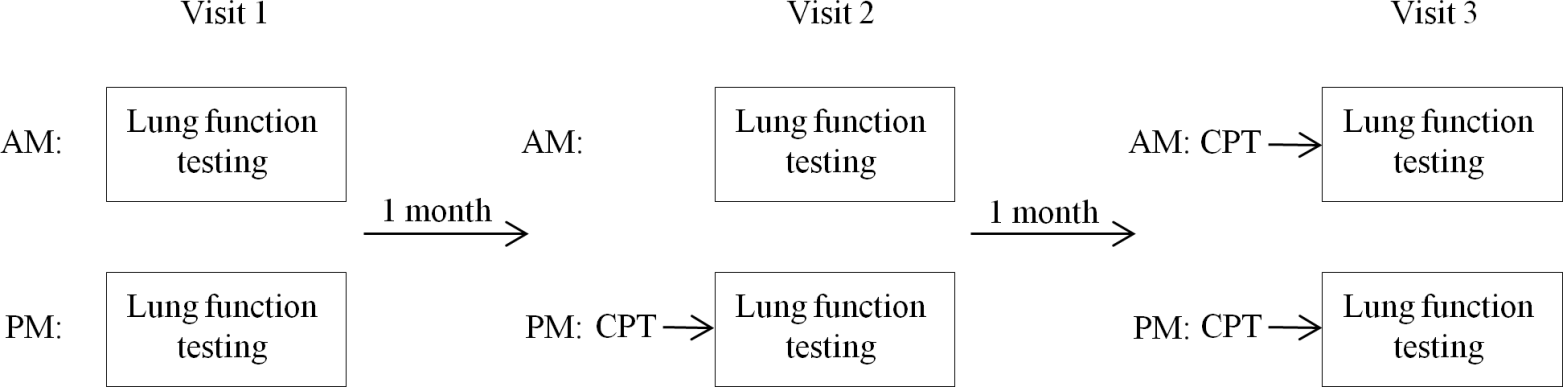
Study design. Lung function testing was performed morning (AM) and afternoon (PM) at each visit. Chest physiotherapy (CPT) was performed immediately prior to afternoon measurements at visit 2 and prior to both morning and afternoon measurements at visit 3.

This was a single-centre prospective non-randomized intervention study that was completed–from inclusion of the first patient until last visit for the last patient–between 1^st^ of March and 15^th^ of August 2013. N_2_MBW, followed by spirometry and plethysmography, were performed in each child at 3 visits that were 1 month apart. These tests were performed both in the morning (between 8:30 AM and 11 AM) and approximately 4 hours later in the afternoon (Fig 2). Half an hour of supervised CPT was scheduled as follows:

Visit 1: no CPT in the morning or in the afternoon prior to measurements. Visit 2: no CPT in the morning; CPT immediately prior to the afternoon measurements. Visit 3: CPT immediately prior to both morning and afternoon measurements. The patients were instructed not to perform CPT or to use bronchodilators at home on the day of the study visits.

The study design allowed the evaluation of both daytime variation and the effect of CPT on MBW measures in the same study. Thus, half of the measurements were performed in the morning and half after CPT.

### Chest physiotherapy

All patients performed 30 minute programs that included 10 minutes of treadmill exercise followed by 15–20 minutes use of the patients’ habitual positive expiratory pressure (PEP) device, huffing, and other airway clearance techniques. CPT did not include inhalation of bronchodilators or mucus-releasing medicine. PEP was performed in blocks of 15 breaths with a mid-expiratory pressure between 10–20 cmH_2_O. Each block was followed by a short session of coughing and forced expiration techniques performed in an upright sitting position. A trained CF physiotherapist (LP) ensured optimal performance and supervised each CPT session.

### Multiple breath nitrogen washout

Primary outcomes were LCI and other N_2_MBW measures. N_2_MBW was performed in accordance with the ERS/ATS consensus statement[13]. We used an open circuit bypass flow system (*Exhalyzer D*, *Eco Medics AG*, *Duernten*, *Switzerland*) and the device-associated software (*Spiroware® version 3*.*1*.*6*, *Eco Medics AG*) for data acquisition and calculation. The participants performed N_2_MBW in an upright sitting position while wearing a nose clip and breathing through a mouthpiece with a tight seal. The estimated pre-capillary dead space, including the bacterial filter, was 35 mL.

The calculation of MBW measures has been described in detail elsewhere[14]. We determined LCI; M_1_/M_0_, moment ratios 1; M_2_/M_0_, moment ratios 2; FRC_MBW_, functional residual capacity calculated from MBW; CEV, cumulative expired volume; S_acin_·VT, the concentration normalized phase III slope of first breath (minus the convection-dependent contribution to this slope) and S_cond_·VT, the concentration normalized phase III slope increase between turnover 1.5 and 6. Software-associated reference data[15] were used to normalize the N_2_MBW measures.

### Spirometry and plethysmography

Secondary outcomes were spirometric and plethysmographic measures. Spirometry and plethysmography were performed in accordance with the ATS/ERS guidelines[16,17] using Jaeger Master Screen Pro (*CareFusion*, *Hoechberg*, *Germany*). The Global Lung Function Initiatives reference equation [18] were used to normalize spirometry parameters. We assessed FEV_1_, forced expired volume in 1 second; FVC, forced vital capacity; FEF_25-75_, forced expiratory flow at 25–75% of FVC; TLC, total lung capacity; FRC_pleth_, functional residual capacity from plethysmography/intrathoracic gas volume; VC, vital capacity; RV, residual volume and RV%TLC, RV/TLC ratio·100%. Additionally, we calculated the difference between FRC derived from plethysmography and MBW (ΔFRC_pleth-MBW_) which is an estimate of trapped gas[19], since non-ventilated lung units are included in the FRC_pleth_ calculation_,_ but not in FRC_MBW_.

### Statistics

For baseline characteristics, the upper limit of normal for the N_2_MBW measures was defined as z-score > 1.96; for spirometry, z-score < -1.64 was defined as abnormal. The sample size calculation was based on previous LCI data from our department (unpublished data) showing a between-subject variance of 2.75 and an intra-individual variation of 0.7 between monthly visits in clinically stable children with CF. Hence, we defined a change in LCI > 0.7 during the day as being clinically significant. As in previous studies[4–6], we expected CPT to have a greater impact on LCI and defined a clinically meaningful effect by CPT as a change in LCI > 1[20]. The calculation was based on the study design in Fig 2 and on the use of a linear mixed model with a random intercept for each child (with a variance of 2.75) and an interaction between child and visit to account for the nested design. We expected a reduction in the intra-individual variation (observed to 0.7 as mentioned above) when measuring the patients twice rather than once a day. The intra-individual variation was set to 0.5 in the calculation and the variance of the interaction term between child and visit was therefore set to 0.2. Time-of-day and CPT were considered fixed effects.

With 8 children in the study, the power to detect the effect of time-of-day and CPT on LCI with a type I error rate of 0.05 was estimated by simulation to be 0.81, accounting for multiple testing using the Benjamini-Hochberg method[21]. We included 10 children in this study assuming the dropout rate to be 20%.

Each outcome measure was analysed using the linear mixed model used for the power calculation except that age was included in the models as a fixed effect since a significant association with age was found for most measures. Sex was not associated with any of the measures and was therefore not included in the models. The residual variance remained stable across visits, which was assessed graphically by plotting residuals against visits. In line with the power calculation, the p-values corresponding to the effect of time-of-day and CPT were corrected for multiple testing using the Benjamini-Hochberg method[21] for each measure separately. The p-values for the effect of time-of-day and CPT for each of the secondary outcomes were not further corrected for the multiplicity arising from testing multiple measures and therefore, we do not focus on p-values of borderline significance for these measures. A model without any fixed effects and a random effects structure as described above was used to determine the overall means and standard deviations (SD) of the measures.

To describe the variation in the measures, we used the linear mixed model to determine the correlation between two measurements and the coefficient of reproducibility (CR) defined as 1.96 times the SD of the difference between two measurements (presented in S1 Table). The correlation and CR were determined comparing measurements taken at the same as well as on different visits (within visit and between visits).

The power calculation was performed using R version 3.2.0 and SAS version 9.4 for the linear mixed models. P-values less than 0.05 were considered significant.

### Ethics

All participants and their parents or guardians provided written informed consent prior to participation. The Danish National Committees on Biomedical Research Ethics for the Capital Region of Copenhagen provided ethical approval (Protocol No. H-1-2010-042).

To view the study protocol, please see: http://dx.doi.org/10.17504/protocols.io.jsjcncn.

## Results

Of the 10 recruited subjects, 2 were excluded from the analysis, 1 because of small continuous leaks during the MBW measurements that were detected during subsequent quality control assessment and 1 due to permanent exacerbation after enrolment. Of the remaining 8 patients (2 boys and 6 girls), the second study visit of 1 patient was excluded from the analysis because of an exacerbation detected retrospectively (spirometric exacerbation criteria). Accordingly, 46 N_2_MBW measurements were available for analysis (43 triplets and 3 doublets). Only one patient had the first visit postponed by 1 month due to an exacerbation.

The patient’s baseline characteristics are presented in Table 1.

**Table 1:** Baseline characteristics of the participants (n = 8) at the first morning study visit.

Parameter	Median (range)	Median z-score (range)
Age (years)	9.6 (6.0; 15.1)	
Weight (kg)	27.0 (21.2; 54.4)	
Height (cm)	134.0 (113.2; 167.5)	
LCI	9.0 (7.1; 13.0)	8.9 (2.2; 23.1)^a^
M_1_/M_0_	1.9 (1.6; 2.9)	8.0 (2.9; 24.5)^a^
M_2_/M_0_	8.2 (5.7; 19.0)	11.3 (4.3; 41.3)^a^
FRC_MBW_ (L)	1.3 (0.9; 2.8)	NA^a^
CEV (L)	11.6 (6.0; 36.7)	NA^a^
S_acin_·VT	0.131 (0.087; 0.197)	6.6 (3.0; 12.2)^a^
S_cond_·VT	0.054 (0.022; 0.116)	8.2 (0.2; 23.8)^a^
FEV_1_ (L)	1.6 (1.4; 3.1)	-0.2 (-1.8;1.4)^b^
FEV_1_ (% predicted)^b^	97.5 (78.5; 117.9)	
FVC (L)	1.9 (1.6; 4.0)	0.1 (-0.7; 1.4)^b^
FVC (% predicted)^b^	100.7 (92.0; 117.7)	
FEF_25-75_ (L/s)	1.9 (1.3; 4.0)	-0.3 (-2.1; 0.3)^b^
FEF_25-75_ (% predicted)^b^	86.5 (54.6; 109.3)	

*Abbreviations*: LCI, lung clearance index; M_1_/M_0_, moment ratios 1; M_2_/M_0_, moment ratios 2; FRC_MBW_, functional residual capacity calculated from MBW; CEV, cumulative expired volume; S_acin_·VT, the concentration normalized phase III slope of first breath (minus the convection-dependent contribution to this slope); S_cond_·VT, the concentration normalized phase III slope increase between turnover 1.5 and 6; FEV_1_, forced expired volume in 1 second; FVC, forced vital capacity; FEF_25-75_, forced expiratory flow at 25–75% of FVC. NA = No available reference data. Reference equations

^a^software-associated reference data[15] and

^b^Quanjer et al.[18].

**Fig 3:**
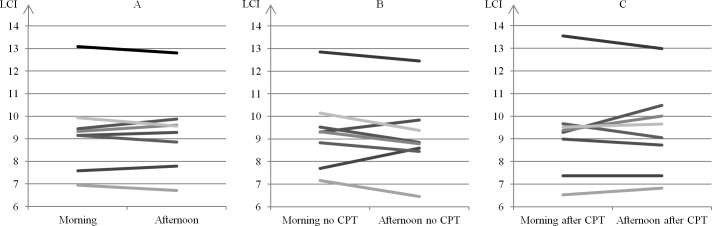
Difference in LCI between morning and afternoon. Lines represent each individual. N = 8. Average of the morning LCI’s compared to the afternoon LCI’s (Fig 3A), average of the two morning LCI’s with no prior CPT compared to the one afternoon LCI with no prior CPT (Fig 3B), and the one morning LCI with prior CPT compared to the average of the two afternoon LCI’s with prior CPT (Fig 3C).

**Fig 4:**
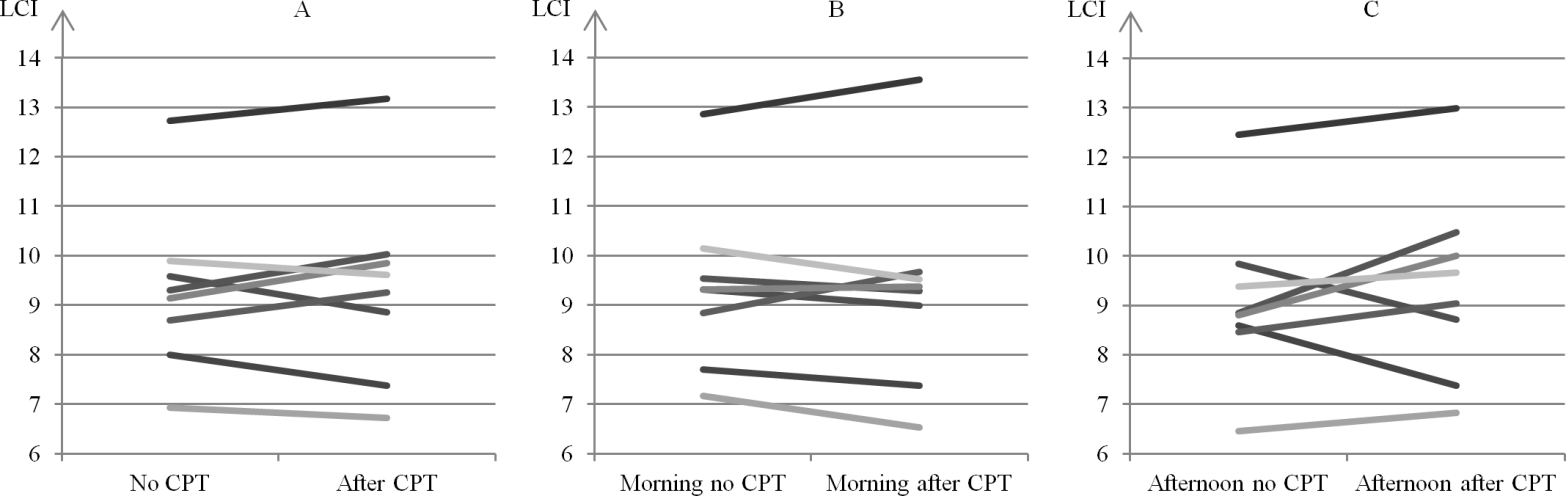
Difference in LCI between measurements with no prior CPT compared to measurements with prior CPT. Lines represent each individual. N = 8. Average of LCI’s with no prior CPT compared to LCI’s with prior CPT (Fig 4A), average of the two morning LCI’s with no prior CPT compared to the one morning LCI with prior CPT (Fig 4B), the one afternoon LCI with no prior CPT compared to the average of the two afternoon LCI’s with prior CPT (Fig 4C).

The LCI and moment ratios were markedly increased at baseline, when all patients had abnormal values. In comparison, most patients had normal spirometric measures that indicated mild to moderate CF lung disease. The median time difference (range) between N_2_MBW measurements in the morning versus afternoon was 3:51 hours (3:10; 4:09), and all patients successfully completed the planned CPT program. As shown in Figs 3 and 4, we observed no consistent changes in the LCI during the day (morning versus afternoon) or between measurements with no prior CPT versus measurements with prior CPT.

In (Fig 3A, 3B and 3C), the difference in the LCI between morning and afternoon (median [range]) were -0.1 (-0.4; 0.4), -0.5 (-0.8; 0.9), and 0.1 (-0.6; 1.2). Out of the 24 lines, the LCI decreased in 13 and increased in 11. A numeric change in LCI > 0.7 was observed in 4 cases (2 increases, 2 decreases) and for 4 different subjects (no trend for specific subjects). In (Fig 4A, 4B and 4C), the differences in the LCI after CPT were (median [range]): 0.1 (-0.7; 0.7), -0.3 (-0.6; 0.8), and 0.4 (-1.2; 1.6). Out of the 24 lines, the LCI decreased in 11 and increased in 13. A numeric change in LCI > 1 was observed in 4 cases (2 increases, 2 decreases) and for 4 different subjects (no trend for specific subjects).

Table 2 shows the effect of time-of-day and CPT on N_2_MBW and spirometry along with the overall mean values (SD) calculated from all visits. Results for plethysmographic measures are presented in S1 Table.

**Table 2:** The overall mean values and the estimated effect of time-of-day (Day) and chest physiotherapy (CPT) on N_2_MBW and spirometry.

Outcome	Overall mean (SD)^a^	Effect estimate^b^ (95% CI)	p-value	Corrected p-value^c^
LCI	9.32 (1.85)	Day: -0.05 (-0.32; 0.22)	0.72	0.72
		CPT: +0.08 (-0.26; 0.42)	0.63	0.72
M_1_/M_0_	2.07 (0.38)	Day: +0.00 (-0.07; 0.07)	0.95	0.95
		CPT: +0.02 (-0.07; 0.10)	0.65	0.95
M_2_/M_0_	9.43 (4.0)	Day: -0.33 (-0.92; 0.26)	0.29	0.57
		CPT: +0.19 (-0.61; 0.99)	0.65	0.65
FRC_MBW_ (L)	1.54 (0.63)	Day: -0.02 (-0.06; 0.01)	0.22	0.44
		CPT: +0.02 (-0.04; 0.07)	0.56	0.56
CEV (L)	15.20 (9.61)	Day: -0.34 (-0.83; 0.16)	0.20	0.35
		CPT: +0.32 (-0.34; 0.98)	0.35	0.35
S_acin_·VT	0.140 (0.052)	Day: +0.003 (-0.015; 0.022)	0.72	0.72
		CPT: +0.006 (-0.019; 0.030)	0.67	0.72
S_cond_·VT	0.050 (0.027)	Day: -0.005 (-0.010; 0.001)	0.13	0.27
		CPT: -0.000 (-0.007; 0.007)	0.96	0.96
FEV_1_ (L)	1.99 (0.81)	Day: -0.00 (-0.05; 0.05)	0.96	0.96
		CPT: -0.03 (-0.11; 0.05)	0.41	0.82
FVC (L)	2.39 (0.96)	Day: -0.00 (-0.05; 0.05)	0.96	0.96
		CPT: -0.01 (-0.06; 0.08)	0.76	0.96
FEF_25-75_ (L/s)	2.11 (1.00)	Day: +0.04 (-0.09; 0.16)	0.57	0.57
		CPT: -0.15 (-0.34; 0.03)	0.11	0.23

*Abbreviations*: LCI, lung clearance index; M_1_/M_0_, moment ratios 1; M_2_/M_0_, moment ratios 2; FRC_MBW_, functional residual capacity calculated from MBW; CEV, cumulative expired volume; S_acin_·VT, the concentration normalized phase III slope of first breath (minus the convection-dependent contribution to this slope); S_cond_·VT, the concentration normalized phase III slope increase between turnover 1.5 and 6; FEV_1_, forced expired volume in 1 second; FVC, forced vital capacity; FEF_25-75_, forced expiratory flow at 25–75% of FVC. N = 8. Lung function test (N_2_MBW and spirometry) from 46 visits overall. SD: standard deviation. 95% CI: 95% confidence interval. +/- = increase/decrease during the day or after chest physiotherapy (CPT).

^a^: Overall means and standard deviations are determined from a linear mixed model without any fixed effects and a random effects structure including a random intercept for each child as well as a random intercept between child and visit.

^b^: All estimates are derived from a linear mixed model including time-of-day (Day) and CPT as fixed effects and the same random effects structure as in ^a^.

^c^: For each outcome, the two p-values for Day and CPT, respectively, are corrected for multiple testing using the Benjamini-Hochberg method[21].

We found no significant daytime differences in the LCI or in the other MBW measures, nor did we find that CPT had a significant effect on the MBW measures. Likewise, we found no effect of time-of-day and CPT on spirometric measures. Exploring the association between time-of-day and CPT on the multiple secondary measures, we found a significant (p<0.01) decrease in FRC_pleth_ (110 mL) during the day, whereas a borderline significant (p = 0.046) decrease in ΔFRC_pleth-MBW_ (90 mL) during the day and a borderline significant (p = 0.03) increase in TLC (110 mL) after CPT was observed, as reported in S1 Table. For most measures, the correlation between measurements taken at different visits was only slightly larger than the correlation between measurements taken at the same visits (see S1 Table). The correlation between LCI measurements performed at the same visit and between different visits was estimated to 0.92 and 0.88, respectively.

## Discussion

The present study is the first to assess daytime variation in MBW measures in patients with CF. It also provides data on the effect of CPT on N_2_MBW values in a cohort of school children with mild to moderate clinically stable CF. In this study, the time-of-day as well as CPT had no significant effect on N_2_MBW measures, suggesting that there is no considerable or consistent variation in N_2_MBW measures during the day or after CPT in clinically stable school children with CF. The study also presents data on the effects of time-of-day and CPT on spirometry and plethysmography (for plethysmographic results; see S1 Table).

### Daytime variation

In this pilot study, we found no clinical meaningful effect of time-of-day on any of the N_2_MBW measures. Lung function testing was performed at various times in the morning and again 4 hours later in the afternoon. We observed heterogeneous responses in the LCI values, but a minimal and non-significant overall decrease in the LCI (95% CI) of -0.05 (-0.32; 0.22) in 4 hours. Changes in LCI > 0.7 was observed in only 4 of the 24 lines in Fig 3.

In a previous study by Amin et al.[22], MBW measurements were performed at baseline, 1, 2, 4 and 24 hours after inhalation of hypertonic vs. isotonic saline. In agreement with our results, they found no change in the LCI level (average change from baseline to follow-up measurements; +0.02 (standard error = 0.10)) in the isotonic saline group.

We had speculated that improvements in mucus clearance during the day would result in improvements in several measures, including the LCI, but this is not what we observed. Several factors might explain this. Nightly mucus stagnation that affects pulmonary function might be minimal in clinically stable CF patients, and any stagnated mucus might be mobilized within the first minutes or hours after awakening (i.e. before our measurements). Furthermore, heterogeneous changes in VI during the day might have affected our findings. It is possible that prolonging the time interval between measurements would result in different observations.

There are currently no recommendations as to the time-of-day that MBW should be performed or whether measurements should be performed at consistent times. Our results indicate that such restrictions are unnecessary in school children with clinically stable CF. However, the present study is limited by its small sample size. Since potential daytime variation could affect both research results and the clinical surveillance of CF lung function, and since this has not been investigated in previous studies, we encourage additional larger studies in the future.

#### Secondary outcomes

We found no effect of time-of-day on spirometric measures. To our knowledge, only one previous study has reported on daytime variation of spirometry[23]. In that study, Weller et al. investigated the effect of CPT on spirometric measures. On a control visit with no CPT-interventions a significant improvement in FEV_1_ and FVC of 9.3 and 5.1% predicted, respectively, was observed from 9:30AM to 4:30PM. We cannot explain these different findings, but it may be argued that spirometric daytime variation could be present in CF patients with less well controlled lung disease in contrast to well controlled children with CF since, indeed, the patients in Weller et al.’s study had significantly lower pulmonary function at baseline than our patients (FEV_1_ 76.5 vs. 97.5% predicted and FVC 89.0 vs. 100.7% predicted).

For plethysmographic measures we observed a statistically significant, but small decrease in FRC_pleth_ (110 mL). To our knowledge no previous studies have assessed daytime variation of plethysmographic measures. The decrease in FRC_pleth_ is physiologically difficult to explain. The borderline decrease in ΔFRC_pleth-MBW_ (90 mL) could indicate that trapped gas decreased during the day, by recruitment of non-ventilated lung units, e.g. due to mucus clearance. However, this was caused by a significant decrease in FRC_pleth_ and not an increase in FRC_MBW_, which would otherwise support this finding.

### Chest physiotherapy

The aim of this study regarding CPT was to assess the short-term effects of CPT on MBW measures in school children with clinically stable CF. Importantly, this study was not designed to evaluate the efficacy of CPT and does not add any information about the long-term effects or general benefits of CPT in CF. In this context, we found no effect of vigorous and extensive CPT performed immediately prior to measurements on LCI or on other N_2_MBW measures.

To our knowledge, only 3 previous studies have reported specifically on the short-term effects of CPT on MBW[4–6] in children with CF. The results are inconsistent but related to age, pulmonary function, and the clinical condition of the study population. Supporting our findings, Fuchs et al.[4] performed a randomized controlled parallel group study in a school-age cohort similar to ours and demonstrated that CPT (flutter or PEP without bronchodilators) preceding SF_6_MBW did not affect LCI or FRC_MBW_. Pfleger et al.[5] examined a mixed CF population that included both children and adults and patients that were close to discharge after an intravenous antibiotic treatment course. They did not find consistent or significant changes in LCI and FRC_MBW_ as measured before and 30 minutes after CPT (including PEP and bronchodilators). However, considerable heterogeneous LCI changes ≥ 1 were observed in 17 of 29 patients after CPT. In our study, clinical meaningful LCI changes ≥ 1 were only observed in 4 out of the 24 lines presented in Fig 4. Thus, heterogeneous responses to CPT were clearly less pronounced in the present study. In a recent study by Grosse-Onnebrink et al.[6] that included both children and adults, improvements in LCI were observed after CPT (high-frequency chest wall oscillation) in 15 of 20 patients who were hospitalized with infectious pulmonary exacerbation in the intervention group. The diverse results may be explained by differences in age, pulmonary function, disease severity, CPT programs (e.g. the inclusion of bronchodilators) and especially clinical conditions (hospitalized with pulmonary infection vs. clinically stable). In addition, everyday adherence to home CPT may be important. We encourage additional larger studies in the future and suggest that they look at the effect of CPT on MBW measures in clinically stable patients, since MBW is primarily used as a disease-monitoring tool.

We did not find CPT to have an effect on MBW measures, which could potentially be explained by several factors. VI in clinically stable children with CF may not be caused by mucus that can be mobilized during CPT but rather by permanent structural changes and/or inflammation in the peripheral airways. Individual responses to CPT, such as mild bronchospasms, could explain the heterogeneous changes in the MBW measures. Moreover, CPT and mucus clearance may lead to re-ventilation of previously non-ventilated lung areas, which again could lead to unchanged, better, or worse overall VI.

It is unclear whether the time between CPT and MBW measurement was optimal in this study, since few published studies have addressed this issue. In a small study by Mortensen et al.[9] significant radioaerosol clearance from the peripheral lung zones was not detected until 1 hour after CPT compared to a control visit. On the other hand, the effect of CPT on spirometry in children with CF appears to be greatest immediately after it is performed[24]. How the timing of CPT prior to measurements might affect VI as assessed by MBW remains unclear. This need to be considered in future studies and may in part explain the lack of a clinical meaningful effect of CPT on MBW measures as observed in this study.

#### Secondary outcomes

We did not find an effect of CPT on the measures of spirometry. Supporting our results, previous studies in CF patients report no[4,11,25,26] or only small [5,10] changes in FEV1 and FVC within a few hours after CPT. As mentioned above, the effect of CPT on spirometry in children with CF appears to be greatest immediately after it is performed[24]. In this study spirometry was performed approximately 30–40 minutes after CPT because of preceding and time consuming MBW. Timing of CPT prior to spirometry could thus, possibly have been more optimal for maximal effect on spirometric measures, but supported by previous findings it seems that immediate effects of CPT on spirometric measures may generally be limited and difficult to detect. The primary role of CPT in stable patients with CF, may thus, be to *prevent* obstruction of peripheral airways thus preventing micro-atelectases, as suggested by Lannefors et al.[27].

For plethysmographic measures, a borderline significant increase in TLC (110 mL) after CPT was observed, whereas remaining measures were unaffected by both time-of-day and CPT. Supporting our findings, two previous studies[5,28] have demonstrated that all static lung volumes were unaffected after PEP-treatment.

### Strengths and weaknesses

In this study, lung function testing was performed at various time points in the morning and 4 hours later in the afternoon. This time interval was chosen to assess potential variation between morning and afternoon measurements in an outpatient setting. Comparable time differences between the two measurements were achieved with a median time difference (range) of 3:51 hours (3:10; 4:09). Prolonging this time interval or performing the measurements at a more consistent, early time point might have increased daytime variation. Nevertheless, we consider the chosen time interval of 4 hours sufficient to potentially affect lung function and due to the practical execution of this study we had to accept variation in the time of the morning and afternoon measurements.

All measurements were performed in a very standardized manner using an extensive CPT program. PEP followed by forced expiration techniques and huffing is an efficient way to achieve sputum clearance and mobilization[9–11]. The CPT program in the present study far exceeds that performed daily by the patients themselves. Hence, it is reasonable to expect that CPT would have a greater impact on N_2_MBW measures in this study than under normal circumstances.

This study only included clinically stable children with CF to better reflect an outpatient setting. The effect of time-of-day and CPT may be different in hospitalized patients with increased mucus obstruction or in patients with more severe or advanced lung disease. Most patients in this study had normal FEV_1_ indicating mild to moderate CF like visits were postponed one month in case of symptoms indicating a respiratory tract infection, for which reason we consider the variance caused by disease state and infection to be small. Furthermore, seasonal variation may play a role. However, we consider it unlikely that season would have a major impact on daytime variation and the effect of CPT in this population with stable CF. Due to these considerations we did not assess the impact of disease state, infection or season in the statistical model, like we do not believe that we have the power to detect any such differences in means and/or variances due to the small sample size.

Based on previous data, we defined the expected effect estimates on LCI as 0.7 and 1.0 for the time-of-day and after CPT, respectively. Despite the calculated power of 81%, the greatest limitation of this study was the ambitious effect estimates resulting in a small sample size. Future studies with larger samples could allow the detection of smaller changes during the day or after CPT.

### Clinical relevance

In a clinical context, N_2_MBW is still in its early stages. It is important to know about factors that influence measures when evaluating the potential role of N_2_MBW as a disease-monitoring tool in a clinical setting as well as in a research setting. In CF, lung function parameters are measured frequently and at various time points. Furthermore, most patients are expected to perform CPT at least twice daily. The present study suggests that the time-of-day as well as the short-term effect of CPT may play minor roles when N_2_MBW is used to estimate both global (LCI and moment ratios) and specific (SnIII indices) VI in clinically stable children with CF. Our study is limited by the small number of patients and the relatively ambitious effect estimates. For this reason, our results are predominantly applicable to routine clinical disease surveillance. It seems very unlikely that the small changes in MBW measures observed in this study due to daytime variation and CPT would influence the treatment approach in routine clinical MBW measurements. However, the cumulative effect of the potentially small effects of various external factors on MBW measures could be critical in clinical research. For this reason, we emphasize that further studies of the effect of both daytime variation and the effect of CPT on MBW measures are warranted.

## Conclusion

In conclusion, this study demonstrated that the time-of-day as well as CPT performed immediately prior to N_2_MBW had no consistent or significant influence on N_2_MBW measures. However, we emphasize that further studies of the effect of both daytime variation and the effect of chest physiotherapy on MBW measures are warranted.

## Supporting information

S1 Table The overall mean values and the estimated effect of time-of-day (Day), chest physiotherapy (CPT) and age (Age) on N_2_MBW, spirometry and plethysmography. Significant effects marked with * and presented in bold. Furthermore, the intercept, the estimated variance parameters, the correlation between two measurements and the coefficient of reproducibility (CR) defined as 1.96 times the SD of the difference between two measurements are shown.
(DOCX)

S1 RawData Excel file with raw data (anonymous) in accordance with PLOS ONEs data availability policy for small datasets. (XLSX)

S1 Abbreviations A list of abbreviations used in this article. (DOCX)

S1 Trial Study Protocol Danish Relevant parts: This is the Danish version of a sub study in the Ph.D. protocol”Ventilation distribution as an early marker of lung disease in children with cystic fibrosis and primary ciliary dyskinesia”. The sub study is a methodological study titled”Influence of chest physiotherapy on MBW in CF” (point 1.1.3. in the Ph.D.-protocol). All parts related or relevant for the mentioned/present sub study from the original Ph.D.-protocol are mentioned. The parts from the original Ph.D.-protocol that has no relevance for the mentioned/present study *and* study protocols and related results that have not yet been published are censured/removed here.
(DOC)

S1 Trial Study Protocol English Relevant parts: This is an English translation of a sub study in the Ph.D. protocol”Ventilation distribution as an early marker of lung disease in children with cystic fibrosis and primary ciliary dyskinesia”. The sub study is a methodological study titled”Influence of chest physiotherapy on MBW in CF” (point 1.1.3. in the Ph.D.-protocol). All parts related or relevant for the mentioned/present sub study from the original Ph.D.-protocol are translated. The parts from the original Ph.D.-protocol that has no relevance for the mentioned/present study *and* study protocols and related results that have not yet been published are censured/removed here.
(DOC)
