# Supplementary material for: The effect of time-of-day and chest physiotherapy on multiple breath washout measures in children with clinically stable cystic fibrosis
Source: PLoS One. 2018 Jan 10;13(1):e0190894. doi: 10.1371/journal.pone.0190894 (PMC5761951; doi:10.1371/journal.pone.0190894)
Supplement: S1 Table — Significant effects marked with * and presented in bold. Furthermore, the intercept, the estimated variance parameters, the correlation between two measurements and the coefficient of reproducibility (CR) defined as 1.96 times the SD of the difference between two measurements are shown. (DOCX) [file pone.0190894.s001.docx]

Supporting information

| **S1 Table. The overall mean values and the estimated effect of time-of-day (Day), chest physiotherapy (CPT) and age (Age) on N_2_MBW, spirometry and plethysmography. Significant effects marked with * and presented in bold. Furthermore, the intercept, the estimated variance parameters, the correlation between two measurements and the coefficient of reproducibility (CR) defined as 1.96 times the SD of the difference between two measurements are shown.** | | | | | | | | |
| --- | --- | --- | --- | --- | --- | --- | --- | --- |
| Outcome | Overall mean (SD)^a^ | Effect estimate^b^ (95% CI) | Unadjusted p-value | Adjusted p-value^c^ | Intercept | Variance of random effects | Correlation | Coefficient of reproducibility |
| LCI | 9.32 (1.85) | Day: -0.05 (-0.32; 0.22)  CPT: +0.08 (-0.26; 0.42)  Age: +0.38 (0.03; 0.73) | 0.72  0.63  0.08 | 0.72  0.72  0.08 | 5.38 | ID: 2.11  ID*Visit: 0.10  Residual: 0.18 | WV: 0.92  BV: 0.88 | WV: 1.19  BV: 1.49 |
| M_1_/M_0_ | 2.07 (0.38) | Day: +0.00 (-0.07; 0.07)  CPT: +0.02 (-0.07; 0.10)  Age: +0.08 (0.00; 0.15) | 0.95  0.65  0.08 | 0.95  0.95  0.08 | 1.29 | ID: 0.09  ID*Visit: 0.01  Residual: 0.01 | WV: 0.88  BV: 0.83 | WV: 0.31  BV: 0.37 |
| M_2_/M_0_ | 9.43 (4.0) | Day: -0.33 (-0.92; 0.26)  CPT: +0.19 (-0.61; 0.99)  Age: +0.81 (0.07; 1.56) | 0.29  0.65  0.08 | 0.57  0.65  0.08 | 0.85 | ID: 9.31  ID*Visit: 0.80  Residual: 0.87 | WV: 0.92  BV: 0.85 | WV: 2.58  BV: 3.58 |
| FRC_MBW_ (L) | 1.54 (0.63) | Day: -0.02 (-0.06; 0.01)  CPT: +0.02 (-0.04; 0.07)  **Age: +0.18 (0.10; 0.26)** | 0.22  0.56  **<0.01*** | 0.44  0.56  **<0.01*** | -0.30 | ID: 0.111  ID*Visit: 0.005  Residual: 0.003 | WV: 0.97  BV: 0.93 | WV: 0.16  BV: 0.25 |
| CEV (L) | 15.20 (9.61) | Day: -0.34 (-0.83; 0.16)  CPT: +0.32 (-0.34; 0.98)  **Age: +2.47 (1.02; 3.93)** | 0.20  0.35  **0.01*** | 0.35  0.35  **0.01*** | -10.69 | ID: 38.22  ID*Visit: 0.45  Residual: 0.62 | WV: 0.98  BV: 0.97 | WV: 2.18  BV: 2.87 |
| S_acin_·VT | 0.140 (0.052) | Day: +0.003 (-0.015; 0.022)  CPT: +0.006 (-0.019; 0.030)  Age: +0.001 (-0.009; 0.011) | 0.72  0.67  0.89 | 0.72  0.72  0.89 | 0.136 | ID: 0.001  ID*Visit: 0.001  Residual: 0.001 | WV: 0.70  BV: 0.46 | WV: 0.08  BV: 0.11 |
| S_cond_·VT | 0.050 (0.027) | Day: -0.005 (-0.010; 0.001)  CPT: -0.000 (-0.007; 0.007)  Age: +0.005 (0.000; 0.010) | 0.13  0.96  0.12 | 0.27  0.96  0.12 | 0.000 | ID: 0.00043  ID*Visit: 0.00003  Residual: 0.00009 | WV: 0.84  BV: 0.79 | WV: 0.03  BV: 0.03 |
| FEV1 | 1.99 (0.81) | Day: -0.00 (-0.05; 0.05)  CPT: -0.03 (-0.11; 0.05)  **Age: +0.24 (0.17; 0.32)** | 0.96  0.41  **<0.01*** | 0.96  0.82  **<0.01*** | -0.58 | ID: 0.08  ID*Visit: 0.01  Residual: 0.01 | WV: 0.94  BV: 0.82 | WV: 0.21  BV: 0.38 |
| FVC | 2.39 (0.96) | Day: -0.00 (-0.05; 0.05)  CPT: -0.01 (-0.06; 0.08)  **Age: +0.30 (0.23; 0.36)** | 0.96  0.76  **<0.01*** | 0.96  0.96  **<0.01*** | -0.72 | ID: 0.07  ID*Visit: 0.01  Residual: 0.01 | WV: 0.93  BV: 0.85 | WV: 0.22  BV: 0.32 |
| FEF_25-75_ | 2.11 (1.00) | Day: +0.04 (-0.09; 0.16)  CPT: -0.15 (-0.34; 0.03)  **Age: +0.26 (0.11; 0.40)** | 0.57  0.11  **0.01*** | 0.57  0.23  **0.01*** | -0.65 | ID: 0.34  ID*Visit: 0.06  Residual: 0.03 | WV: 0.92  BV: 0.79 | WV: 0.51  BV: 0.83 |
| TLC (L) | 3.31 (1,32) | Day: -0.06 (-0.13; 0.01)  **CPT: +0.11 (0.02; 0.20)**  **Age: +0.40 (0.30; 0.50)** | 0.12  **0.02***  **<0.01*** | 0.12  **0.046***  **<0.01*** | -0.87 | ID: 0.17  ID*Visit: 0.01  Residual: 0.01 | WV: 0.94  BV: 0.88 | WV: 0.30  BV: 0.41 |
| FRC_pleth_ (L) | 1.63 (0.70) | **Day: -0.11 (-0.18; -0.05)**  CPT: +0.05 (-0.03; 0.13)  **Age: +0.20 (0.14; 0.27)** | **0.00***  0.25  **<0.01*** | **<0.01***  0.25  **<0.01*** | -0.51 | ID: 0.07  ID*Visit: 0.01  Residual: 0.01 | WV: 0.88  BV: 0.80 | WV: 0.29  BV: 0.37 |
| VC (L) | 2.36 (0.95) | Day: -0.01 (-0.06; 0.05)  CPT: +0.05 (-0.03; 0.13)  **Age: +0.30 (0.24; 0.35)** | 0.82  0.26  **<0.01*** | 0.82  0.52  **<0.01*** | -0.75 | ID: 0.05  ID*Visit: 0.01  Residual: 0.01 | WV: 0.88  BV: 0.72 | WV: 0.25  BV: 0.38 |
| RV (L) | 0.94 (0.43) | **Day: -0.08 (-0.15; -0.01)**  CPT: +0.07 (-0.03; 0.17)  **Age: +0.11 (0.05; 0.17)** | **0.03***  0.18  **<0.01*** | 0.07  0.18  **<0.01*** | -0.21 | ID: 0.05  ID*Visit: 0.02  Residual: 0.01 | WV: 0.86  BV: 0.61 | WV: 0.29  BV: 0.48 |
| RV%TLC | 28.91 (5.31) | Day: -1.57 (-4.16; 1.03)  CPT: +0.02 (-2.58; 2.62)  Age: -0.47 (-1.35; 0.40) | 0.25  0.99  0.33 | 0.49  0.99  0.33 | 33.09 | ID: 9.94  ID*Visit: -^d^  Residual: 18.34 | WV: -^d^  BV: 0.35 | WV: -^d^  BV: 11.87 |
| ΔFRC_pleth-MBW_ (L) | 0.09 (0.20) | **Day: -0.09 (-0.15; -0.02)**  CPT: +0.03 (-0.05; 0.12)  Age: +0.03 (-0.01; 0.06) | **0.01***  0.45  0.17 | **0.03***  0.45  0.17 | -0.23 | ID: 0.02  ID*Visit: 0.01  Residual: 0.01 | WV: 0.71  BV: 0.48 | WV: 0.29  BV: 0.38 |
| N = 8. Lung function test (N_2_MBW, spirometry and plethysmography) from 46 visits overall. SD: standard deviation. 95% CI = 95% confidence interval. +/- = in­crease/decrease during the day, after CPT and per year of age, respectively. ID: the variance of the random subject-specific intercept. ID*Visit: The variance of the random interaction between subject and visit. Residual: The variance of the residual error term. WV: within visits. BV: between visits. Significant differences in bold and marked with *.  ^a^: Overall means and standard deviations are determined from a linear mixed model without any fixed effects and a random effects structure including a random intercept for each child as well as a random intercept between child and visit.  ^b^: All estimates are derived from a linear mixed model including time-of-day (Day) and CPT as fixed effects and the same random effects structure as in ^a^.  ^c^: For each outcome, the confidence intervals and the two p-values for Day and CPT are adjusted for multiple testing using the Benjamini-Hochberg method.  ^d^: Not estimable.  *Abbreviations*: LCI, lung clearance index; M_1_/M_0_, moment ratios 1; M_2_/M_0_, moment ratios 2; FRC_MBW_, functional residual capacity calculated from MBW; CEV, cumulative expired volume; S_acin_·VT, the concentration normalized phase III slope of first breath (minus the convection-dependent contribution to this slope); S_cond_·VT, the concentration normalized phase III slope increase between turnover 1.5 and 6; FEV_1_, forced expired volume in 1 second; FVC, forced vital capacity; FEF_25-75_, forced expiratory flow at 25–75% of FVC; TLC, total lung capacity; FRC_pleth_, functional residual capacity from plethysmography/intrathoracic gas volume; VC, vital capacity; RV, residual volume; RV%TLC, RV/TLC ratio·100%; ΔFRC_pleth-MBW_, the difference between FRC derived from plethysmography and from N_2_MBW. | | | | | | | | |
