# Supplementary material for: The effect of time-of-day and chest physiotherapy on multiple breath washout measures in children with clinically stable cystic fibrosis
Source: PLoS One. 2018 Jan 10;13(1):e0190894. doi: 10.1371/journal.pone.0190894 (PMC5761951; doi:10.1371/journal.pone.0190894)
Supplement: S1 Abbreviations — (DOCX) [file pone.0190894.s003.docx]

S1 Abbreviations: a list of abbreviations used in this article.

MBW, multiple breath washout; N_2_MBW, multiple breath nitrogen washout; VI, ventilation distribution inhomogeneity; CF, cystic fibrosis; CPT, chest physiotherapy; PEP, positive expiratory pressure.

MBW measures: LCI, lung clearance index; M_1_/M_0_, moment ratios 1; M_2_/M_0_, moment ratios 2; FRC_MBW_, functional residual capacity calculated from MBW; CEV, cumulative expired volume; S_acin_·VT, the concentration normalized phase III slope of first breath minus the convection-dependent contribution to this slope; S_cond_·VT, the concentration normalized phase III slope increase between turnover 1.5 and 6.

Spirometric measures: FEV_1_, forced expired volume in 1 second; FVC, forced vital capacity; FEF_25-75_, forced expiratory flow at 25–75% of FVC.

## Plethysmographic measures: TLC, total lung capacity; FRC_pleth_, functional residual capacity from plethysmography/intrathoracic gas volume; VC, vital capacity; RV, residual volume; RV%TLC, RV/TLC ratio · 100%; ΔFRC_pleth-MBW_, the calculated difference between FRC derived from plethysmography and MBW, respectively, which is an estimate of trapped gas, since non-ventilated lung units are included in the FRC_pleth_ calculation_,_ but not in FRC_MBW_.
