# Supplementary material for: The effect of time-of-day and chest physiotherapy on multiple breath washout measures in children with clinically stable cystic fibrosis
Source: PLoS One. 2018 Jan 10;13(1):e0190894. doi: 10.1371/journal.pone.0190894 (PMC5761951; doi:10.1371/journal.pone.0190894)
Supplement: S1 Trial Study Protocol Danish — The sub study is a methodological study titled”Influence of chest physiotherapy on MBW in CF” (point 1.1.3. in the Ph.D.-protocol). All parts related or relevant for the mentioned/present sub study from the original Ph.D.-protocol are mentioned. The parts from the original Ph.D.-protocol that has no relevance for the mentioned/present study and study protocols and related results that have not yet been published are censured/removed here. (DOC) [file pone.0190894.s004.doc]

This is the danish version of the substudy in the Ph.D. protocol ”Ventilation distribution as an early marker of lung disease in children with cystic fibrosis and primary ciliary dyskinesia”. The substudy is a methodological study titled ”Influence of chest physiotherapy on MBW in CF” (point 1.1.3. in the Ph.D.-protocol). All parts related or relevant for the mentioned/present substudy from the original Ph.D.-protocol are mentioned. The parts from the original Ph.D.-protocol that has no relevance for the mentioned/present study *and* study protocols and related results that have not yet been published are censured/removed here. These deleted parts are mentioned with a comment which are marked with yellow highlight.

The original Ph.D.-protocol in Danish is attached, but needs to be handled as confidential. If further details are required, please let us know.

Ventilationsdistribution som tidlig markør for lungeskade hos børn med cystisk fibrose og primær ciliedyskinesi

Projektansvarlig: Kent Green, cand. med.^1^

Hovedvejleder: Kim G. Nielsen, overlæge, dr. med.^1^

Medvejledere: Tacjana Pressler, overlæge, dr. med.^1^

Per Gustafsson, overlæge, docent, ph.d.^4^

Frederik Buchvald, afdelingslæge, ph.d.^1^

Samarbejdspartnere: Niels Høiby, professor, overlæge, dr. med.^2^

Karen Damgaard, overlæge, dr. med.^3^

June Kehlet Marthin, cand. med., ph.d.^1^

Birgitte Hanel, bioanalytiker, dr. med.^1^

^1^Pædiatrisk klinik I, Juliane Marie Centret

^2^Klinisk mikrobiologisk afdeling, Diagnostisk Center

^3^Diagnostisk radiologisk klinik, Diagnostisk Center

Rigshospitalet

^4^Børneafdelingen, Skaraborg Sjukhus, Skövde, Sverige

Sted: Dansk BørneLunge Center (DBLC)

Pædiatrisk klinik I, Juliane Marie Centret

Rigshospitalet

Resumé

Påvisning og behandling af lungeskade tidligt i forløbet antages at være afgørende for prognosen ved de medfødte og kroniske progressive lungesygdomme Cystisk Fibrose (CF) og Primær Cilie Dyskinesi (PCD). Sekretstagnation og sekundært infektioner og inflammation i luftvejene medfører allerede fra spædbarnsalderen tiltagende lungeskade, som er grundlæggende for tidlig død ved CF og reduceret lungefunktion ved PCD. Tidligere påvisning af igangværende lungeskade vil give mulighed for tidligere intervention af intensiv behandling, forbedret overvågning, og yderligere forbedring af den forebyggende behandling. Dette vil øge livskvaliteten ved begge sygdomme, og med stor sandsynlighed overlevelsen ved CF.

Lungefunktionen er en vigtig indikator for prognose og behandlingseffekt, men de aktuelt anvendte målemetoder registrerer ikke de tidligste lungeskader hos skolebørn og er pga. krav til kooperation uanvendelige til spæd- og småbørn. Der er derfor behov for en mere sensitiv metode som kan bruges i alle aldersgrupper. Gasudvaskning - Multiple Breath inert gas Washout (MBW) - er en teknik, som opfylder disse krav og giver store forventninger til påvisning af minimale lungeskader længe før symptomgennembrud og inden andre metoder giver information herom.

MBW er for første gang etableret i Danmark under Dansk BørneLungeCenter på Rigshospitalet, hvor et nyoprettet BørneRespirationsFysiologisk Laboratorium tilbyder en lang række metoder til vurdering af lungesygdom hos børn.

Part regarding “background” removed since it is not specific to the present study.

Formålet med dette projekt er at undersøge:

- Pålidelighed af resultater udført ved MBW under forskellige standardiserede omstændigheder (reproducerbarhed), herunder etablering af dansk normalmateriale for børn.
- Hvorledes MBW kan bruges som markør for tidlig lungeskade sammenlignet med konventionelle lungefunktionsmålingsmetoder ved de kroniske lungesygdomme CF og PCD.
- Hvorledes MBW kan anvendes som effektparameter, når der interveneres i CF basis behandling.
- Part regarding a larger substudy censured – since this is confidential and not published yet.

Projektet vil generere nye og mere aggressive kontrol- og behandlingsregimer med det overordnede mål at reducere sygeligheden samt forbedre livskvaliteten og overlevelsen hos patienter med CF og PCD. Desuden forventes projektet at frembringe viden, som kan bruges indenfor de væsentligt hyppigere kroniske lungesygdomme astma og KOL, som rammer 10% og 15% af henholdsvis børn og voksne.

Part regarding “material and methods” removed since it is not specific for the present study and since the same and further detail are provided in the following.

1. **METODOLOGISK STUDIE**

Substudy 1.1.1 and 1.1.2. removed since it is not relevant for the present study.

- 1. **Indflydelse af lungefysioterapi på MBW ved CF:**

1.1.3.1. Deltagere: 10 børn med cystisk fibrose.

1.1.3.2. Inklusionskriterier: Positiv svedtest og/eller tilstedeværelsen af to CF mutationer. Alder 5-18 år.

1.1.3.3. Eksklusionskriterier: Pågående øvre eller nedre luftvejsinfektion. Fuchs kriterier >2.

1.1.3.4. Primære endpoints: Absolutte værdier af LCI, Scond og Sacin.

1.1.3.5. Sekundære endpoints:

- FEV_1_ og (se ovenfor) sRaw
- Andel af vækst af mikroorganismer i luftvejssekret.

1.1.3.6. Procedure - 3 besøg med 1 måneds interval imellem hvert besøg.

I. besøg: Kl. 10 udføres 3 MBW med god teknik uden foregående lungefysioterapi. Dvs. deltager må ikke have udført PEP-maske behandling eller deltaget i hård fysisk anstrengelse samme morgen.

Kl. 14. udføres 3 MBW med god teknik. Deltager må i mellemtiden ikke have udført lungefysioterapi eller deltaget i hård fysisk anstrengelse.

II. besøg: Kl. 10 udføres 3 MBW med god teknik uden foregående lungefysioterapi. Dvs. deltager må ikke have udført PEP-maske behandling eller deltaget i hård fysisk anstrengelse samme morgen.

Kl. 14. udføres på ny 3 MBW med god teknik forudgået af lungefysioterapi indenfor 1 time vejledt af fysioterapeut.

III. besøg: Kl. 10 udføres 3 MBW med god teknik forudgået af lungefysioterapi indenfor 1 time vejledt af fysioterapeut.

Kl. 14. udføres på ny 3 MBW med god teknik forudgået af lungefysioterapi indenfor 1 time vejledt af fysioterapeut.

- Luftvejssekret tages fra ved hvert besøg til mikrobiologisk undersøgelse.
- Ved hvert besøg foretages indledningsvist klinisk vurdering med symptomscoring ud fra Fuchs kriterier(26). Deltager stetoskoperes og der tages vitale parametre: respirationsfrekvens, puls, blodtryk perifer saturationsmåling.

1.1.3.7. Informeret samtykke: Informeret samtykke vil blive indhentet fra alle patienter og/eller deres forældre (ved alder<15 år).

Substudy 2, 3 and 4 are removed since they have no relevance for the present study and since results have not yet been published.

Udtagelse af biologisk materiale

Luftvejssekret: Som ovenfor beskrevet vil der i nogle af delforsøgene blive taget luftvejssekret fra til mikrobiologisk undersøgelse. Dette vil foregå enten ved ophost af sekret (ekspektorat) eller ved sug igennem næsen (larynxsug), såfremt ophost ikke er muligt. Larynxsug foregår ved, at et tyndt sugekateter føres ind gennem næsen og ned i svælget. Når suget nærmer sig den øverste del af luftrøret, (larynx) vil det typisk fremprovokere hoste, hvorved vi kan få suget slim op, der kommer fra de nedre luftveje. Dette vil blive foretaget ved erfaren sygeplejerske. Der vil blive indsamlet ca. 5 ml luftvejssekret.

Part regarding bloodsamples are removed since no bloodsamples was collected in the present study.

Statistiske metoder

Demografisk data vil blive opgjort som mean/median værdier, standard deviation (SD) og range eller konfidensintervaller afhængigt af normalfordeling.

Reproducerbarhed af de enkelte parametre bestemt ved MBW findes ved udregning af variationskoefficienten (CV) som 100 x SD/mean af de tre enkelte målinger. Variabiliteten findes ved brug af Bland-Altman plot.

For data, som udviser normalfordeling, vil Pearson’s eller Spearman’s test blive brugt til beregning af korrelation mellem forskellige variable. Normalfordelte data testes med t-test. Ikke-normalfordelte data sammenlignes ved brug af non-parametriske tests, parrede. Statiske undersøgelser vil blive udført ved brug af *SAS version. 9.1.3*.

Part regarding results from previous studies has been removed since this is an introduction to the more basic methodological findings, whereas study-specific pervious findings are discussed in the manuscript of the present study.

Perspektiv

LCI måling hos børn og unge med CF og PCD kan på sigt vise sig at være vigtigt supplement til almindelig lungefunktionsmåling og hos børn under 5 år blive en helt ny metode til monitorering af progression af sygdom hos tilsyneladende asymptomatiske patienter. Endelig vil metoden kunne vise sig mere sensitiv til at påvise effekt af eksisterende og potentielle nye behandlingstilbud. Sådanne tiltag har tidligere vist sig at kunne bedre overlevelse og prognose. Endvidere vil resultaterne kunne danne basis for en bedre monitorering af andre kroniske lungesygdomme, som er væsentlig hyppigere i befolkningen, såsom astma og KOL som rammer henholdsvis 10% af børn og 15% og voksne (33-35).

Bivirkninger, risici, ulemper mv.

MBW og de øvrige lungefysiologiske metoder er alle non-invasive undersøgelser uden gener for patienten og alle undersøgelser er i forvejen fast rutine, som tilbydes alle patienter i DBLC.

*Part deleted regarding bronchoalveolar lavage, universal anaesthesia and CT-scans, not relevant for current study.*

Der vurderes ikke at være risici forbundet ved udtagelse af biologisk materiale. Dog kan ophentning af larynx sekret ved sugning gennem næsen være forbundet med let ubehag. Ved blodprøve i fingeren kan fingerspidsen som eneste bivirkning blive let øm.

*Part deleted regarding pausation of habitual CF treatment, not relevant for current study.*

Der forventes således på ingen måde væsentlige risici ved deltagelse i nogen af delstudierne og det forsøgsansvarliges vurdering at fordele ved deltagelse i projektet langt overstiger ulemper og evt. bivirkninger.

Etiske aspekter

Hos patienter med CF og PCD bliver indhentning af luftvejssekret gjort rutinemæssigt ved alle kliniske kontakter i ambulatoriet, hvorfor der ikke er tale om ekstra indførte dyrknings undersøgelser. Hvis mikrobiologisk svar sammenholdt med forsøgsdeltagerens tilstand giver indikation for antibiotisk behandling vil deltageren blive sat i relevant behandling, i henhold til ambulatoriets vanlige retningslinier. Indhentning og dyrkning af luftvejssekret vil blive udført efter vanlig procedure, hvor biologisk materiale (sekret) bliver destrueret umiddelbart efter opstart af dyrkning, dvs. indenfor 1-2 dage. Sekretet vil således ikke blive opbevaret i en biobank og da kun bakterier bliver gemt, vil der i projektet ikke blive opbevaret personhenførbart biologisk materiale.

For interventionsdelene er der i henhold til værktøjsundtagelsen ikke tale om et klinisk forsøg med lægemidler på mennesker, idet lægemidlet alene benyttes til afklaring af fysiologiske mekanismer. Af den årsag vurderes anmeldelse til EudracT og Lægemiddelsstyrelsen samt GCP (Good Clinical Practice)-monitorering ikke at være nødvendig.

Der vil i projektet ikke blive gjort brug af placebo- eller kontrolbehandling.

Alle resultater - positive såvel som negative - vil blive sendt ind til publikation i international peer-reviewed tidsskrifter med hel eller delvis fokus indenfor pædiatrisk lungemedicin.

For både raske forsøgsdeltagere samt patienter med CF og PCD vil deltagelse i projektet bidrage til, at MBW kan undersøges som en mulig test til børn og unge til udelukkelse hhv. skærpelse af mistanken om forværring i lungeskade hos dem selv samt andre patienter med samme lungesygdom før symptomer herpå kan konstateres. Tidlig diagnose af lungeskade for børn og unge med CF og PCD er vigtig for, hvor godt disse børn bevarer deres lungefunktion op gennem livet, hvorfor deltagelse vil have stor værdi for begge patientgrupper.

Egen fordel ved deltagelse i projektet for patienter med både CF og PCD er, at han/hun bliver fulgt mere intenst med flere undersøgelser i CF-ambulatoriet på Rigshospitalet, mens projektet står på.

Egen fordel ved deltagelse i projektet for raske forsøgspersoner er, at han/hun i forbindelse med besøget/besøgene i Dansk BørneLunge Center på Rigshospitalet vil blive grundigt lægeundersøgt.

Herudover bidrager forsøgsdeltagerne, raske som patienter, også til at genere ny viden om anvendeligheden af MBW, som på sigt kan være til egen gavn samt evt. være til gavn for patienter med andre lungesygdomme, såsom Kronisk Obstruktiv Lungesygdom (KOL) eller astma.

Det vurderes, at risici og evt. bivirkninger langt opvejes af de forventelige fordele ved deltagelse i forskningsprojektet.

Projektet er godkendt af De Videnskabsetiske Komitéer for Region Hovedstaden med nr: H-1-2010-042.

Projektet er d. 20. maj 2010 godkendt af Datatilsynet.

Informeret samtykke vil blive indhentet fra alle patienter og/eller deres forældre (ved alder < 15 år).

Rekruttering

Rekruttering af raske forsøgsdeltagere vil foregå ved mundtlig forespørgsel samt ved ophæng af annonce på udvalgte skoler og daginstitutioner. Rekruttering af patienter med CF eller PCD vil foregå i forbindelse med de regelmæssige ambulante kontroller, som foregår i DBLC på Rigshospitalet. Forældre og patienter vil blive informeret om muligheden for at få en bisidder med ved informationssamtalen. Interesserede forældre og/eller forsøgspersoner vil herefter blive inviteret til en informationssamtale, hvor de under uforstyrrede forhold og, hvis de ønsker det med tilstedeværelse af bisidder, vil blive grundigt informeret mundtligt og få udleveret skriftlig deltagerinformation. Information til de mindreårige vil blive givet af en person med kendskab til området for projektet, og som desuden har de nødvendige pædagogiske forudsætninger for kommunikation med mindreårige. Informationssamtalen vil foregå i ét af ambulatoriets konsultationslokaler, hvor der er muligheden for at låse af. Forud for indhentning af skriftlige samtykke gives 14 dages betænkningstid. Mundtlig forespørgsel samt efterfølgende mundtlig og skriftlig information vil blive udført af den projektansvarlige eller en personaleperson oplært i projektet af den projektansvarlige.

Informeret samtykke vil blive indhentet fra alle forsøgspersoner og/eller deres forældre (ved alder < 15 år).

Der vil ikke blive givet vederlag til forsøgspersoner. Transportudgifter afholdes af forældrene.

Praktisk gennemførelse

LCI udføres kun ganske få steder i verden, hvilket skyldes det omkostningstunge udstyr. Udstyr er anskaffet for eksterne midler og metoden etableret på DBLC. Undersøgelsen vil blive udført af erfarne bioanalytikere samt den projektansvarlige, som alle har været på studieophold i Sverige (hos Docent Overlæge Per Gustafsson), hvor metoden har været etableret og anvendt gennem flere år. Der er tilknyttet en række forskningserfarne samarbejdspartnere, som alle har publiceret indenfor PCD og CF, således at projektet med stor sandsynlighed kan gennemføres. Ph.d.-projektet kommer til at foregå i et allerede etableret forskningsmiljø, hvorfra der primo 2010 er udgået 2 ph.d.-afhandlinger og hvor alle vejledere/samarbejdspartnere har lang forskningserfaring.

Begge patientpopulationer er unikke og velbeskrevet med hensyn til almindelige kliniske parametre i form af månedlig klinisk kontrol, bakteriologisk undersøgelse og lungefunktionsundersøgelse. Alle kliniske data indsamles prospektivt (løbende) og indtastes i de allerede etablerede kliniske databaser. Både CF og PCD populationerne hører til de største og bedst kontrollerede i verden. Behandlings resultater af CF patienter hører endvidere til de bedste i verden.

DBLC, Pædiatrisk klinik I, Juliane Marie Centret, Rigshospitalet, har landsdelsfunktion for behandling af CF (ca. 300 patienter) samt landsfunktion for diagnostik og behandling af PCD (ca. 100 patienter).

Tidsplan

Forventet varighed fra først inkluderede patient til sidst inkluderede patient er målt færdig: ca. 30 måneder.

Økonomi

Forskningsprojektet er blevet til i samarbejde med forsøgsansvarlige i samarbejde med benævnte hovedvejleder samt medvejledere.

Der er på nuværende tidspunkt tilsagt støtte fra Cystisk Fibrose Foreningen med 3 måneders løn (= 126.498,84 kr.) til projektansvarlige og fra Dr. Louises Børnehospitals Forskningsfond med 70.000 kr. til løn.

Forsøgsansvarlige har ingen økonomisk relation til ovenstående støttegivere. Støtten er blevet udbetalt ved indsættelse på forsøgsansvarliges forskningskonto på Rigshospitalet bundet til indeværende projekt. Dertil er der tilsagt støtte fra Rigshospitalets 1 års introduktionsstipendiat på yderlige 1 års løn sv.t. 505.995,36 kr. med start af udbetaling d. 1. august 2010.

Desuden har Dansk BørneLunge Center, hvor ph.d.-projektet finder sted, garanteret yderligere ½ års løn (= 252.997,68 kr.) til projektansvarlige. Der søges løbende andre fonde og legater til dækning af den resterende del af 3 års løn, således at ph.d.-projektet vil blive gennemført. De Videnskabsetiske Komitéer for Region Hovedstaden vil blive orienteret om navn, beløb og udbetalingsmåde, såfremt der opnås yderligere støtte til projektet.
