# Supplementary material for: The effect of time-of-day and chest physiotherapy on multiple breath washout measures in children with clinically stable cystic fibrosis
Source: PLoS One. 2018 Jan 10;13(1):e0190894. doi: 10.1371/journal.pone.0190894 (PMC5761951; doi:10.1371/journal.pone.0190894)
Supplement: S1 Trial Study Protocol English — The sub study is a methodological study titled”Influence of chest physiotherapy on MBW in CF” (point 1.1.3. in the Ph.D.-protocol). All parts related or relevant for the mentioned/present sub study from the original Ph.D.-protocol are translated. The parts from the original Ph.D.-protocol that has no relevance for the mentioned/present study and study protocols and related results that have not yet been published are censured/removed here. (DOC) [file pone.0190894.s005.doc]

This is an English translation of a substudy in the Ph.D. protocol ”Ventilation distribution as an early marker of lung disease in children with cystic fibrosis and primary ciliary dyskinesia”. The substudy is a methodological study titled ”Influence of chest physiotherapy on MBW in CF” (point 1.1.3. in the Ph.D.-protocol). All parts related or relevant for the mentioned/present substudy from the original Ph.D.-protocol are translated. The parts from the original Ph.D.-protocol that has no relevance for the mentioned/present study *and* study protocols and related results that have not yet been published are censured/removed here. These deleted parts are mentioned with a comment which is marked with yellow highlight.

Ventilation distribution as an early marker of lung disease in children with cystic fibrosis and primary ciliary dyskinesia

Primary investigator: Kent Green, cand. med.^1^

Primary supervisor: Kim G. Nielsen, chief physician, dr. med.^1^

Co-supervisors: Tacjana Pressler, chief physician, dr. med.^1^

Per Gustafsson, chief physician, docent, ph.d.^4^

Frederik Buchvald, senior medical officer, ph.d.^1^

Collaborators: Niels Høiby, professor, chief physician, dr. med.^2^

Karen Damgaard, chief physician, dr. med.^3^

June Kehlet Marthin, cand. med., ph.d.^1^

Birgitte Hanel, medical laboratory technologist , dr. med.^1^

^1^Pediatric Clinic I, ”Juliane Marie Centret”, Copenhagen University Hospital

^2^Clinical Microbiological Department, ”Diagnostisk Center”, Copenhagen University Hospital

^3^Diagnostic Radiologically Clinic, ”Diagnostisk Center”, Copenhagen University Hospital

^4^Pediatrics Department, Skaraborg Hospital, Skövde, Sweden

Site: The Pediatric Pulmonary Service

Pediatric Clinic I, ”Juliane Marie Centret”

Copenhagen University Hospital, Denmark

Summary

It is presumed that early detection and treatment of the chronic lung diseases cystic fibrosis (CF) and primary ciliary dyskinesia (PCD) are crucial for improving the prognosis. Mucus stagnation and secondary infections and inflammation in the airways causes progressive lung damage from infancy in CF and PCD, which again causes early death in CF and reduced pulmonary function in PCD. Early detection of ongoing lung damage will facilitate early intervention with intensive treatment, enhance disease surveillance and further improve preventive treatment. This will improve quality of life for patients with both CF and PCD and likely improve survival in CF.

Pulmonary function is an important marker of prognosis and effect of treatment, but current pulmonary function tests do not register the earliest damages in the lungs in school children – and are inappliccable for infants and toddlers due to requirements for good coorporation. For this reason there is a demand for more sensitive methods that can be used in all age groups. Gas washout – multiple breath inert gas washout (MBW) – is a method that meets these criteria and seems promising for detection of minimal lung damage prior to debut of clinical symptoms and detection by other methods.

MBW is for the first time in Denmark established in the Pediatric Pulmonary Service at Copenhagen University Hospital, where a recently established ”Respiratory Physiology Lab for Children” offers a wide range of methods to detect lung disease in children.

Part regarding “background” removed since it is not specific to the present study.

**Aim:**

The aim of the Ph.D.-project is to investigate:

- Reliability of results conducted from MBW measurements under different standardized conditions (reproducibility), including establishment of a Danish reference material for children.
- Whether MBW can be used as a marker of early lung disease compared to conventional lung function methods in the chronic lung diseases CF and PCD.
- Whether MBW can be used as a relevant outcome to detect interventions in basic CF treatment.
- Part regarding a larger substudy censured – since this is confidential and not published yet.

The Ph.D.-project will generate a new and more aggressive approach to surveillance and treatment of patients with CF and PCD with an overall aim to reduce morbidity and mortality and improve quality of life. Furthermore, it is expected that this project will generate new knowledge that can be used in the more common lung diseases asthma and COPD, which affects 10% and 15% of children and adults, respectively.

Part regarding “material and methods” removed since it is not specific for the present study and since the same and further detail are provided in the following.

1. **METHODOLOGICAL STUDY:**

Substudy 1.1.1 and 1.1.2. removed since it is not relevant for the present study.

- 1. **Influence of chest physiotherapy on MBW in CF:**

1.1.3.1. Participants: 10 children with cystic fibrosis.

1.1.3.2. Inclusion criteria: Positive sweat test and/or the presence of two CF mutations. Age: 5-18 years.

1.1.3.3. Exclusion criteria: Current upper or lower airway infection. Fuchs criteria >2.

1.1.3.4. Primary endpoints: Absolute values for LCI, Scond and Sacin.

1.1.3.5. Secondary endpoints:

- FEV_1_ and sRaw
- Proportion of microorganisms in mucus.

1.1.3.6. Design - 3 visits 1 month apart.

I. visit: 10 AM: 3 technical acceptable MBW measurements with no prior chest physiotherapy. That is: Participants cannot perform chest physiotherapy at home prior to visits, including use of a PEP-mask or participating in strenuous physical activity.

2 PM: 3 technical acceptable MBW measurements with no prior chest physiotherapy. Participants cannot perform chest physiotherapy or participate in strenuous physical activity between measurements.

II. visit: 10 AM: 3 technical acceptable MBW measurements with no prior chest physiotherapy. That is: Participants cannot perform chest physiotherapy at home, prior to visit including use of a PEP-mask or participating in strenuous physical activity.

2 PM: 3 technical acceptable MBW measurements with prior chest physiotherapy supervised by a trained chest physiotherapist performed within 1 hour before measurements.

III. visit: 10 AM: 3 technical acceptable MBW measurements with prior chest physiotherapy supervised by a trained chest physiotherapist performed within 1 hour before measurements.

2 PM: 3 technical acceptable MBW measurements with prior chest physiotherapy supervised by a trained chest physiotherapist performed within 1 hour before measurements.

- At each visit a sample of mucus is collected and sent for microbiological investigation.
- At each visit an initial clinical assessment using the Fuchs criteria is performed. At each visit auscultation of the lungs and vital signs are measured: respiration frequency, heart rate, blood pressure and oxygen saturation.

1.1.3.7. Informed consent: An informed consent will be signed by all participants or their guardians prior to participation.

Substudy 2, 3 and 4 are removed since they have no relevance for the present study and since results have not yet been published.

**Collection of biological material**

Airway mucus: As described above mucus will be collected in some of the substudies for microbiological investigation. Mucus will be collected by expectoration when coughing or by suction through the nose, if expectoration by coughing is not possible. Suction is a method where a narrow catheter is inserted in to a nostril and moved to the pharynx. When the catheter tip is getting glose to the upper part of the larynx it will typically induce coughing, whereby mucus coming from the lower airways can be collected. This will be performed by experienced nurse. Approximately 5 ml of mucus will be collected this way.

Part regarding bloodsamples are removed since no bloodsamples was collected in the present study.

Statistical method

Demographic data will be reported as mean/median values, standard deviation (SD) and range or confidence intervals depending on the normal distribution.

Reproducibility of each MBW measurement is found by calculating the coefficient of variation (CV) as follows: 100 x SD/mean of the 3 MBW runs per visit. The variability is found using Bland-Altman plot.

Pearson’s or Spearman’s test will be used to calculate the correlation between different variables for data that are normal distributed. T-tests are used to test normal distributed data. Non-normal distributed data will be compared using paired non-parametric tests. Statistical analyses will be performed using *SAS version. 9.1.3*.

Part regarding results from previous studies has been removed since this is an introduction to the more basic methodological findings, whereas study-specific pervious findings are discussed in the manuscript of the present study.

Perspective

LCI measurements in children and adolescents with CF and PCD may become an important addition to current lung function tests as well as a new method to monitor progression of airway disease in seemingly asymptomatic patients in children below 5 years of age. Furthermore, the method may prove more sensitive than current lung function tests in detecting the effect of existing and potentially upcoming treatments. Similar approaches have previously shown to be able to improve survival and prognosis. Additionally, the results may form the basis for better monitoring of other more common chronic lung diseases like asthma and COPD, which affects 10% and 15% of children and adults, respectively.

Adverse events, risks, disadvantages, etc.

MBW and other physiological lung function tests (performed in this project) are all non-invasive methods with no discomfort for the patients and all methods are already regularly performed or offered to all patients that adhere to the Pediatric Pulmonary Service, Copenhagen University Hospital.

*Part deleted regarding bronchoalveolar lavage, universal anaesthesia and CT-scans, not relevant for current study.*

There are no expected risks related to the collection of biological material (that is) mucus. However, collection of mucus from larynx using suction may cause discomfort. The fingertip may become sore after blood samples taken from the fingertip.

*Part deleted regarding pausation of habitual CF treatment, not relevant for current study.*

Thus, there are no expected risks for the patients participating in any of the substudies and in the opinion of the investigators the advantages of participating in the project exceed the disadvantages and the risk of possible adverse events.

Ethical aspects

Collection of mucus from patients with CF and PCD are routinely performed at all outpatient control visits, and are thus not extraordinarily performed in this project. Patients will be treated with antibiotics according to the department’s normal guidelines according to microbiological test results and/or clinical symptoms. Collection of biological material will be performed according to the department’s usual guidelines, where biological material (mucus) will be destroyed immediately after it has been cultured, that is within 1-2 days. Thus, the mucus will not be stored, only microbiological results will be stored, and no personally identifiable biological material will be stored.

The intervention studies will not be classified as clinical trials according to the rule about trials in which the medical product is used as a tool to investigate physiological responses. For this reason, registration of this protocol in EudracT and in the Danish Medicine Agency as well as GCP (Good Clinical Practice)-monitoring is not deemed necessary.

There will not be used placebo or control treatments in this project.

All results – positive and negative findings – will be submitted for publication in international peer-reviewed journals primarily or partly focused on pediatric pulmonology.

For all participants in the above mentioned studies, including both healthy subjects and subjects with CF and PCD, participation will contribute to clarify whether MBW can be used as a lung functions test in children and adolescents to diagnose and/or to exclude progression in lung disease in the patients themselves and in other patients with the same lung disease before debut of symptoms. Early detection of lung damage in children and adolescents with CF and PCD is important to maintain a stable pulmonary function in later stages of life, and participation in the project will be of great value for the individual patients with both CF and PCD. Participation in the project will likely be an advantage for each patient with either PCD or CF, since he/she will be followed more closely than usual with more examinations and tests at the Pediatric Pulmonary Service at Copenhagen University Hospital as long as he/she is enrolled in the project. It is deemed an advantage for healthy controls participating in the study that he/she will be thoroughly examined by a doctor at the Pediatric Pulmonary Service at Copenhagen University Hospital.

Furthermore, by participating, both healthy controls and patients will help us expand our knowledge about MBW, which prospectively might be an advantage for themselves and other patients with different lung diseases such as asthma or COPD.

It is deemed that possible disadvantages and the risk of possible adverse events are easily compensated by the advantages of participating in this project.

This project was approved by The Danish National Committees on Biomedical Research Ethics for the Capital Region of Copenhagen with the following protocol no: H-1-2010-042.

This project was approved by the Danish Data Protection Agency on 20^th^ of May 2010.

All participants and their parents or guardians will provide a written informed consent prior to participation.

Recruitment

Recruitment of healthy participants will take place by oral request and by posters at selected schools and day care centres. Recruitment of patients with CF or PCD will take place during regular outpatient control visits at the Pediatric Pulmonary Service at Copenhagen University Hospital. Parents and patients will be informed that they can bring a counsellor at the information conversation. Parents and patients who show interest in participation will be invited to an information visit where they will be thoroughly informed orally under quiet conditions with time for questions and receive written information for participants. A person with knowledge about the project and who also possesses the necessary pedagogical communicative skills will inform minors. The information visit will take place in one of the usual consultation rooms at the Pediatric Pulmonary Service at Copenhagen University Hospital with the possibility of locking the door. The written informed consent will not be collected until at least 14 days of reflection time has passed from the information visit.

Oral request and subsequent oral and written information will be performed by the primary investigator or by staff who have been trained in the protocol by the primary investigator.

All participants and their parents or guardians will provide a written informed consent prior to participation.

There will not be given any remuneration to any participants. Payment for transportation will be covered by the parents.

Practical execution

LCI measurements are only performed a few places in the world due to the expensive equipment. Equipment has been purchased by external funds and the method is already established at the Pediatric Pulmonary Service at Copenhagen University Hospital. The measurements will be performed by experienced medical laboratory technologists and by the primary investigator, who have all completed a course in Sweden (at docent chief physician Per Gustafsson), where the method have already been established and applied for many years.

Several experienced scientists, who have published scientific work regarding PCD and CF will act as associated collaborators to enhance the execution of the project. The Ph.D.-project will take place in an already established research environment, where two Ph.D.-theses were completed in 2010, and where all supervisors/collaborators have comprehensive research experience.

Both patient populations are unique and well described in the literature regarding management of clinical control visits, microbiological collection and evaluation of lung functions tests. All clinical data in this project will be collected prospectively and documented in already established databases. The CF and PCD populations at the Pediatric Pulmonary Service at Copenhagen University Hospital are among the largest and clinically most well controlled in the world. Furthermore, the treatment regimen of CF patients at the Pediatric Pulmonary Service at Copenhagen University Hospital is among the best in the world.

The Pediatric Pulmonary Service at Copenhagen University Hospital is responsible for the regional treatment of CF patients in Denmark (approximately 300 patients) as well as diagnostics and treatment of all Danish patients with PCD (approximately 100 patients).

Timeline

The expected duration from first included patient until last visit for last included patient: approximately 30 months.

Economics

This scientific project is established in cooperation between the primary investigator, the mentioned primary supervisor and co-supervisors.

At the moment, the Danish Cystic Fibrosis Association provide financial support with salary for 3 months (=126,498.84 DKK) to the primary investigator. ”Dr. Louises Børnehospitals Forskningsfond” provide financial support with 70,000.00 DKK for salary for the primary investigator.

The primary investigator has no economic relation to the above-mentioned financial supporters. The money has been paid out at the primary investigators research bank account at Copenhagen University Hospital and is bound to this project. Furthermore, Copenhagen University Hospital has funded salary for one year as a ”first year scientific introduction salary” with 505,995.36 DKK with monthly payments starting from the 1^st^ of August 2010.

Additionally, the Pediatric Pulmonary Service at Copenhagen University Hospital, where the Ph.D.-project will take place, have guaranteed an additional ½ years salary (=252,997.68 DKK) to the primary investigator. Other scholarships and funds/foundations will continuously be applied to cover the rest of the 3 years salary, in order to ensure completion of the Ph.D.-project.

The Danish National Committees on Biomedical Research Ethics for the Capital Region of Copenhagen will be informed of any contributions, fund-names, amount and the way of payment, in case further contributions are achieved.
